# TMF/ARA160 Governs the Dynamic Spatial Orientation of the Golgi Apparatus during Sperm Development

**DOI:** 10.1371/journal.pone.0145277

**Published:** 2015-12-23

**Authors:** Yoav Elkis, Shai Bel, Roni Rahimi, Tali Lerer-Goldstein, Smadar Levin-Zaidman, Tatiana Babushkin, Sally Shpungin, Uri Nir

**Affiliations:** 1 The Mina and Everard Goodman Faculty of Life Sciences, Bar-Ilan University, Ramat-Gan, 52900, Israel; 2 Electron Microscopy Unit, Weizmann Institute of Science, Rehovot, 7610001, Israel; The Chinese University of Hong Kong, HONG KONG

## Abstract

TMF/ARA160 is known to be a TATA element Modulatory Factor (TMF). It was initially identified as a DNA-binding factor and a coactivator of the Androgen receptor. It was also characterized as a Golgi-associated protein, which is essential for acrosome formation during functional sperm development. However, the molecular roles of TMF in this intricate process have not been revealed. Here, we show that during spermiogenesis, TMF undergoes a dynamic change of localization throughout the Golgi apparatus. Specifically, TMF translocates from the cis-Golgi to the trans-Golgi network and to the emerging vesicles surface, as the round spermatids develop. Notably, lack of TMF led to an abnormal spatial orientation of the Golgi and to the deviation of the trans-Golgi surface away from the nucleus of the developing round spermatids. Concomitantly, pro-acrosomal vesicles derived from the TMF^-/-^ Golgi lacked targeting properties and did not tether to the spermatid nuclear membrane thereby failing to form the acrosome anchoring scaffold, the acroplaxome, around the cell-nucleus. Absence of TMF also perturbed the positioning of microtubules, which normally lie in proximity to the Golgi and are important for maintaining Golgi spatial orientation and dynamics and for chromatoid body formation, which is impaired in TMF^-/-^ spermatids. *In-silico* evaluation combined with molecular and electron microscopic analyses revealed the presence of a microtubule interacting domain (MIT) in TMF, and confirmed the association of TMF with microtubules in spermatogenic cells. Furthermore, the MIT domain in TMF, along with microtubules integrity, are required for stable association of TMF with the Golgi apparatus. Collectively, we show here for the first time that a Golgi and microtubules associated protein is crucial for maintaining proper Golgi orientation during a cell developmental process.

## Introduction

Spermatogenesis is an intricate developmental process, which leads to sperm development and maturation. This process is comprised of two main phases: i) spermatogenesis, during which spermatogonia undergo successive mitotic divisions to form spermatocytes. These cells then divide meiotically, resulting in round spermatids [1, 2], and ii) spermiogenesis, during which round spermatids undergo morphological changes, thereby maturing through elongated spermatids into spermatozoa, which are adapted for fertilization [2, 3]. These multi-step processes are tightly controlled and are governed by both extrinsic hormonal signals and intricate intrinsic regulatory cascades. The complexity of the system makes it susceptible to various defects that can jeopardize male fertility [4].

Apart from its important roles in somatic cells, the Golgi apparatus is one of the most important organelles involved in the spermiogenesis process [5, 6]. The Golgi apparatus is responsible for the generation and release of pro-acrosomal vesicles derived from its trans-Golgi compartment. These pro-acrosomal vesicles are targeted toward the outer surface of the spermatid nuclear envelope and are held by a cytoskeletal structure termed the acroplaxome [7]. The acroplaxome consists of F-actin and Keratin 5 sheets and externally overlays the nuclear envelope, thereby serving as a scaffold for the pro-acrosomal vesicles [7, 8]. When fused to the acroplaxome the pro-acrosomal vesicles initiate acrosome formation, which upon maturation exhibits lysosomal like characteristics including acidity and localization of lysosomal associated proteins [9]. It was demonstrated that the interaction between the Golgi-derived pro-acrosomal vesicles and the acroplaxome and the fusion of the pro-acrosomal vesicles onto the developing acroplaxome surface, is crucial for acrosome maturation. Failure of attachment of the developing acrosome to the acroplaxome results in major defects in acrosome formation, leading to non-functional sperm that are unable to fertilize ova [8, 10, 11]. The key role of Golgi derived, acroplaxome-targeted vesicles in acrosome formation, is reflected by the spatial tilting around of the Golgi apparatus during spermiogenesis. In somatic cells, the trans-Golgi compartment faces the cytoplasmic cell-membrane, thereby enabling the involvement of the Golgi in both secretion of cargo from the cell through exocytosis of Golgi-derived secretory vesicles [12] and endocytosis and retrograde trafficking of proteins, among other molecules, from the external surface of the cell to the inner-cell compartments [12, 13]. The Golgi exhibits a peri-nuclear localization where it lies in close proximity to the microtubule organizing center (MTOC) and interacts with microtubules [14–16].

Microtubules organization and integrity are crucial for maintaining a functional Golgi architecture in both somatic and spermatogenic cells [17, 18]; these fibers act through Golgi-associated Rab and Kinesin-like proteins, which also interact with microtubules [19, 20]. Notably, in somatic cells the Golgi organelle was shown to affect the microtubule structure via mutual protein interactions [14]. Golgi-derived vesicles are mobilized by cytoskeletal structures, either composed of actin filaments or microtubules, thereby controlling their trafficking from the Golgi to their targets [21]. This targeted mobilization of Golgi-derived vesicles is exerted by a number of motor-proteins that act as mediators between the vesicles surface and cytoskeletal filaments [22]. The common localization, orientation and functioning of the Golgi as seen in somatic cells is modified in in sperm cells during spermiogenesis due to the unique and pivotal role of the Golgi complex in this cellular differentiation process. The Golgi is spatially rotated such that the trans-Golgi compartment faces the spermatid nuclear envelope rather than the outer cell membrane [23, 24]. Although being a crucial step in acrosome formation and sperm maturation the factors and processes regulating this unique spatial rearrangement process have not been revealed.

We have previously shown that the Golgi-associated protein TMF/ARA160 (TMF) controls spermiogenesis through several mechanisms. The protein is required for both acrosome formation and for efficient removal of the cytoplasm from elongated spermatids [3]. TMF is also required for maintaining normal levels of testosterone, which in males, is produced mainly in the endoplasmic reticulum (ER) and mitochondria of Leydig cells [4]. It should be noted that the Golgi apparatus also plays a poorly understood role in the testosterone production and secretion from these cells [4]. These roles of TMF are manifested during the spermiogenic process, and, while acrosome formation is linked to the Golgi-related function of TMF inside spermatogenic cells, the effect on testosterone secretion may be attributed to its presence in other cells in the male reproductive system, such as Leydig cells [4]. Since unlike most other traits [25, 26], TMF^-/-^ spermatids lack any targeting of pro-acrosomal vesicles to the spermatid nuclear envelope, we studied the role of TMF in controlling the functional contribution of the Golgi to the guided generation and trafficking of pro-acrosomal vesicles and acrosome formation. We show here that TMF is essential in governing the altered spatial orientation of the Golgi apparatus and in ensuring the formation of the acroplaxome during the early stages of spermiogenesis, to enable acrosome build up. Thus, TMF is the first reported regulator of dynamic spatial re-orientation of the Golgi during a cellular differentiation process.

## Materials and Methods

All animal experiments and tissues experiments were performed according to the guidelines of the Institutional Animal Care and Use Committee of the Bar-Ilan University, Israel. Approval number 28062012.

### Animals

All animal experiments were performed according to the guidelines of the Institutional Animal Care and Use Committee of the Bar-Ilan University, Israel. Approval number 28062012. Mice were housed five per cage with unlimited access to food and water and exposure to 12h light, 12h dark cycles. No manipulations causing suffering to the animals were used during this study. A total number of fifty, 10–15 weeks old male mice were used for this study. Mice of identical ages were selected in pairs from each genotype (wt and TMF^-/-^) to minimize the variability among animals. Male wild type (wt) and TMF^-/-^ ICR mice were obtained by inter-crossing. Heterozygous male mice were inter-crossed with TMF^-/-^ female mice to generate homozygous tmf null offspring. Genotypes were determined by PCR analysis of 0.5 centimeter tail-piece DNA, using primers corresponding the tmf gene and lacZ as previously described [3].

For tissues extraction, a painless method of animals sacrifice was adopted using either CO2 or cervical dislocation.

### Preparation of spermatogenic cell suspension

To isolate spermatogenic cells, mice testes were removed and digested in isolation medium (DMEM with 2 mM glutamine, 1.5 mM sodium pyruvate, 10% fetal calf serum, 25 μg/ml Ampicillin and 1×non-essential amino acids) supplemented with 2 mg/ml collagenase (Sigma-Aldrich) for 5 min at 37°C, and washed twice in isolation medium. Next, isolation medium supplemented with 1 unit/ml DNAse, and 0.3 ml 0.5% trypsin was added per 1ml of isolation medium and the cells were incubated for 5 min at 37°C. The cells were then filtered through a nylon mesh with 50 μm pores. The dissociated cells were deposited on microscope slides for further analyses.

### Immunocytochemistry of spermatogenic and NIH3T3 cells

Spermatogenic cells were spread on Superfrost plus (Superfrost ^®^ Plus, Thermo scientific, USA) glass slides. NIH3T3 cells were seeded (1X 10^6^) in 6 well plates, and grown on a cover glass for 24h. Spermatogenic or NIH3T3 cells were fixed with 4% paraformaldehyde for 30 min followed by 1h incubation with a permeabilization solution containing 0.1% Triton X-100 (Sigma-Aldrich, Israel) in PBS, at room temperature. After permeabilization, nonspecific reactive sites were blocked with PBS-containing 10% fetal bovine serum for 1 hour at room temperature. The cells were then incubated overnight (O.N) at 4°C with either anti-GM130 (1:100, Abcam, USA), anti-tubulin (1:100, Abcam, USA) anti-KIF17B (1:100, Abcam, USA), anti Golgin 97 (1:100, Abcam, USA), anti 58K (1:100, Sigma, USA) or anti TGN38/46 (1:100, Abcam, USA). Cells were washed three times (5 min) in PBS containing 0.2% Tween-20 (Sigma-Aldrich, Israel) to remove non-specifically bound antibody. Bound primary antibodies were detected by Alexa Fluor^®^ 488 goat anti—mouse IgG antibody or Alexa Fluor^®^ 594 goat anti—rabbit IgG antibody (Molecular Probes, Invitrogen, Paisley, U.K.). Nuclei were visualized by incubation with Hoechst 33342 solution for 10 min (1:500). The immuno-stained slides were inspected under an AxioimagerZ1 (Zeiss, Germany) fluorescence microscope, or a confocal fluorescence microscope (Olympus-FV1000). The exact spermiogenic stages of the inspected spermatids were determined using a phase contrast microscopy. Nonspecific staining, determined by incubation with secondary antibody, without primary antibody, was negligible. The presented images represent one out of four independent immuno-staining experiments which yielded similar results.

### Acrosomal and actin filament staining in mouse spermatids

Sperm cells were spread on microscope slides. After air drying, the cells were either fixed in 4% paraformaldehyde for 30 min, or exposed to hypotonic shock to remove the cytoplasm organelles as previously described [7] and then fixed in 4% paraformaldehyde for 30 min. After fixation, the cells were washed twice in PBS for 5 min each. For acrosomal staining, the sperm cells were incubated with PNA-FITC (Sigma-Aldrich 25 μg/ml in PBS) for 30 min followed by one wash for 10 min in DDW, and mounted with FluoroGuard Antifade (BioRad Lab., Richmond, CA). Fluorescent staining of sperm actin filaments was carried out as described before, using phalloidin 594 (Biotium, USA) (Cohen et al., 2004). Each image represents one out of twenty different cells selected from three different sections which gave similar results.

### Transmission electron microscopy (TEM)

Testes were removed from the mice and immediately immersed in Karnovsky fixative (2.5% glutaraldehyde, 2.5% paraformaldehyde in cacodylate buffer pH 7.4) for O.N incubation. The samples were then washed in 0.1 M cacodylate buffer and post-fixed with 1% OsO4 in 0.1 M sodium cacodylate buffer (Sigma-Aldrich, Israel) for 1 h. Samples were then dehydrated in alcohol and propylene oxide followed by embedding in Agar Mix. Thin sections (60 nm) were cut, stained with uranyl acetate and lead citrate, and observed with a FEI Tecnai transmission electron microscope (TEM). Each image represents one out of twenty different cells selected from five different sections which gave similar results

### Immunogold labeling and electron microscopy analysis

Testes were fixed in 4% paraformaldehyde with 0.1% glutaraldehyde in 0.1M cacodylate buffer (pH = 7.4) for 1 hour at room temperature, and kept at 4°C for no longer than 2 days. The samples were soaked overnight in 2.3M sucrose and rapidly frozen in liquid nitrogen. Frozen ultrathin (70–90 nm) sections were cut with a diamond knife at −120°C on a Leica EM UC6 ultramicrotome. The sections were collected on 200-mesh Formvar coated nickel grids. Sections were blocked with a solution containing 1% BSA, 0.1% glycine, 0.1% gelatin, and 1% Tween 20. Immuno-labeling was performed using affinity purified anti-TMF antibodies (1:50, Sigma, Israel), overnight at 4°C, followed by exposure to goat anti-Rabbit IgG coupled to 10-nm gold particles (1:20, Jackson Immunoresearch, USA), for 30 min at room temperature. Contrast staining and embedding were performed as previously described [27]. The embedded sections were viewed and photographed with a FEI Tecnai SPIRIT (FEI, Eidhoven, Netherlands) transmission electron microscope operated at 120 kV, and equipped with an EAGLE CCD Camera. Nonspecific staining, determined by incubation with secondary antibody, without primary antibody, was negligible. Each image represents one out of twelve different cells selected from three different sections which gave similar results.

### Quantification and Statistical analysis of immuno-gold staining

Golgi sub-compartments were identified according to their morphology and vesicles budding. Each Golgi section was inspected for positive immuno-gold staining of TMF. Five different stained cells residing at the same spermatogenic stage and selected from three different sections were screened and their positive signals were quantified.

Statistical analysis was performed using the paired and unpaired Student’s t-test, with a P value <0.05 being considered significant. Results are depicted as mean ± standard deviation (SD) of the mean for n given samples.

### *In-silico* analysis of TMF domains

Murine TMF aa sequence was analyzed for new putative domains using the "Simple Modular Architecture Research Tool" (SMART, http://smart.embl-heidelberg.de/) algorithm. This algorithm enables the identification of known functional domains that are contained in a given protein sequence. To allow an unbiased and informative analysis of the TMF aa sequence the first aa of the TMF protein sequence was replaced to avoid the identification of TMF by the program, as a known protein comprised of previously identified motifs.

### Co-Immunoprecipitation (co-IP)

Protein lysates (1mg) were prepared from tissues of adult mice, as described [4] and incubated with protein A Sepharose (GE Healthcare) for 1 h at 4°C to avoid nonspecific binding of proteins to the beads. The protein lysates were then incubated overnight at 4°C with 1:100 diluted antibodies. Antigen—antibody complexes were precipitated with protein A Sepharose (GE Healthcare) after overnight incubation at 4°C. Precipitates were washed twice with PBS buffer (NaCl 137 mM, KCl 2.7 mM, Na_2_HPO_4_ 10 mM, KH_2_PO_4_ 1.8 mM). Immune complexes were eluted from the Sepharose beads by 10 min incubation in sample buffer (2% SDS, 1% DTT 0.02% bromo-phenol blue 10% glycerol, 12.5mM EDTA, 50 mM Tris-HCl pH 6.8) followed by 8 min incubation at 100°C. The eluted precipitates were separated on SDS-PAGE. The proteins were visualized by western blotting using specific antibodies. The presented immunoblots represent one out of five independent co-IP experiments which yielded similar results.

### Immunoblot analysis

Protein lysates were prepared for western blot analysis, as described [4]. Affinity-purified anti-TMF antibodies (1:1000, Sigma, USA) were used to detect the TMF protein by western blot (WB) analysis. Tubulin, golgin97 and Hexokinase I (HK I) levels were detected using anti-tubulin (DSHB, USA, 1:1000), anti- HK I (Cell Signaling, USA, 1:1000) and anti-golgin97 (Abcam, USA, 1:1000) antibodies.

### Precipitation of microtubules

Microtubules precipitation was performed as previously described [28, 29]. In brief, spermatogenic cells were extracted from adult mice testes as described above and incubated with 4 μM Taxol or 10μM Colchicine for 10 min at room temperature. Cells were then lysed by being suspended for 10 min at room temperature in a microtubules stabilizing buffer (0.1 M Pipes, pH 6.9, 2 M glycerol, 5 mM MgCl2, 2 mM EGTA, 1% Triton X-100, and protease inhibitors) supplemented with either 4 μM Taxol to maintain microtubules stability, or with 10 μM Colchicine to allow microtubules disassembly for negative control. The supernatant containing solubilized proteins was clarified by centrifugation (20,000 *g* for 45 min, 37°C) and was separated from the pellet containing sedimented polymerized microtubules. The pellet was washed once in microtubules-stabilizing buffer before being suspended in a denaturation sample buffer. The samples were subjected to a WB analysis for determining the association of TMF with the sedimented microtubules. The presence of both HK I and golgin97 proteins in the supernatant but not in the microtubules precipitate was used as a negative control. The presented immunoblot represent one out of four independent precipitation experiments which yielded a similar result.

### Cell culture, Colchicine treatment and cell transfection

NIH3T3 cells were grown in Dulbecco's modified Eagle's medium (DMEM) (Biological Industries, Israel) supplemented with 12% fetal bovine serum (FBS) (Biological Industries, USA). Regions of interest from *tmf/ara160* were amplified by PCR reaction as described before [3] using the appropriate primers described in S1 Table. These amplified DNA sequences were cloned into pIRES-EGFP plasmids. Plasmids containing the *tmf/ara160* sequences fused to *gfp* were transiently transfected into NIH3T3 cells using the LipofectAMINE 2000 reagent, according to the manufacturer's instructions (Invitrogen, USA), and as previously described [30]. The transfected cells were treated or untreated with 10μM Colchicine (Sigma-Aldrich, Israel) to allow microtubules disturbance [18] and were taken for immunocytochemistry analysis, as described above. The presented images represent one out of four different transfection and immuno-staining experiments which yielded similar results.

## Results

### Dynamic distribution of TMF in the Golgi compartment of developing round spermatids

**Fig 1:**
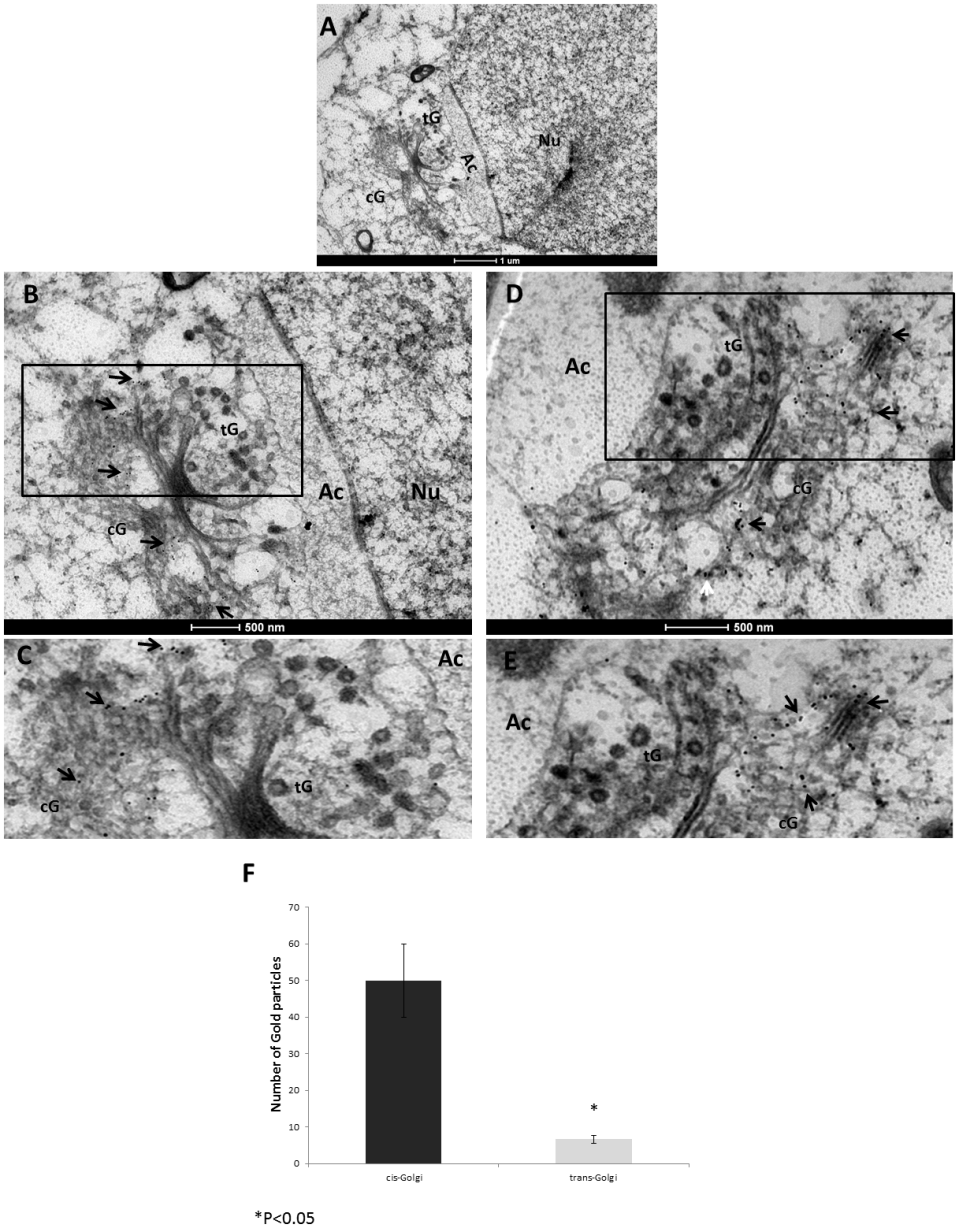
TMF resides in the cis-Golgi of stages 3–4 round spermatids. Testicular 80 nm thick sections from 10 weeks old wt mice were subjected to IG-EM analysis using anti-TMF antibodies followed by an anti-rabbit secondary antibody conjugated to gold particles with a diameter of 10 nm (black dots). (A) Stage 4 spermatids. (B) The same spermatid from **A** under higher magnification, showing the staining for TMF in the peripheral cis-Golgi (indicated by arrows). (C) Enlarged boxed area in **B**. TMF staining is marked by arrows. (D) A late stage 3 spermatid showing the same peripheral cis-Golgi localization of TMF. (E) Enlarged Boxed area in **D**. TMF positive staining is marked by arrows. cG = cis-Golgi, tG = trans-Golgi, Nu = Nucleus, Ac = acrosome. Bars represent 1μm (A), and 500nm (B-E). Each image represents one out of twelve different cells selected from three different sections which gave similar results. (F) Comparative quantification of the immuno-gold labeled TMF in the different Golgi compartments. n = 5 (different cells of the same stage from three different sections), histograms represent mean values +/- SD.

**Fig 2:**
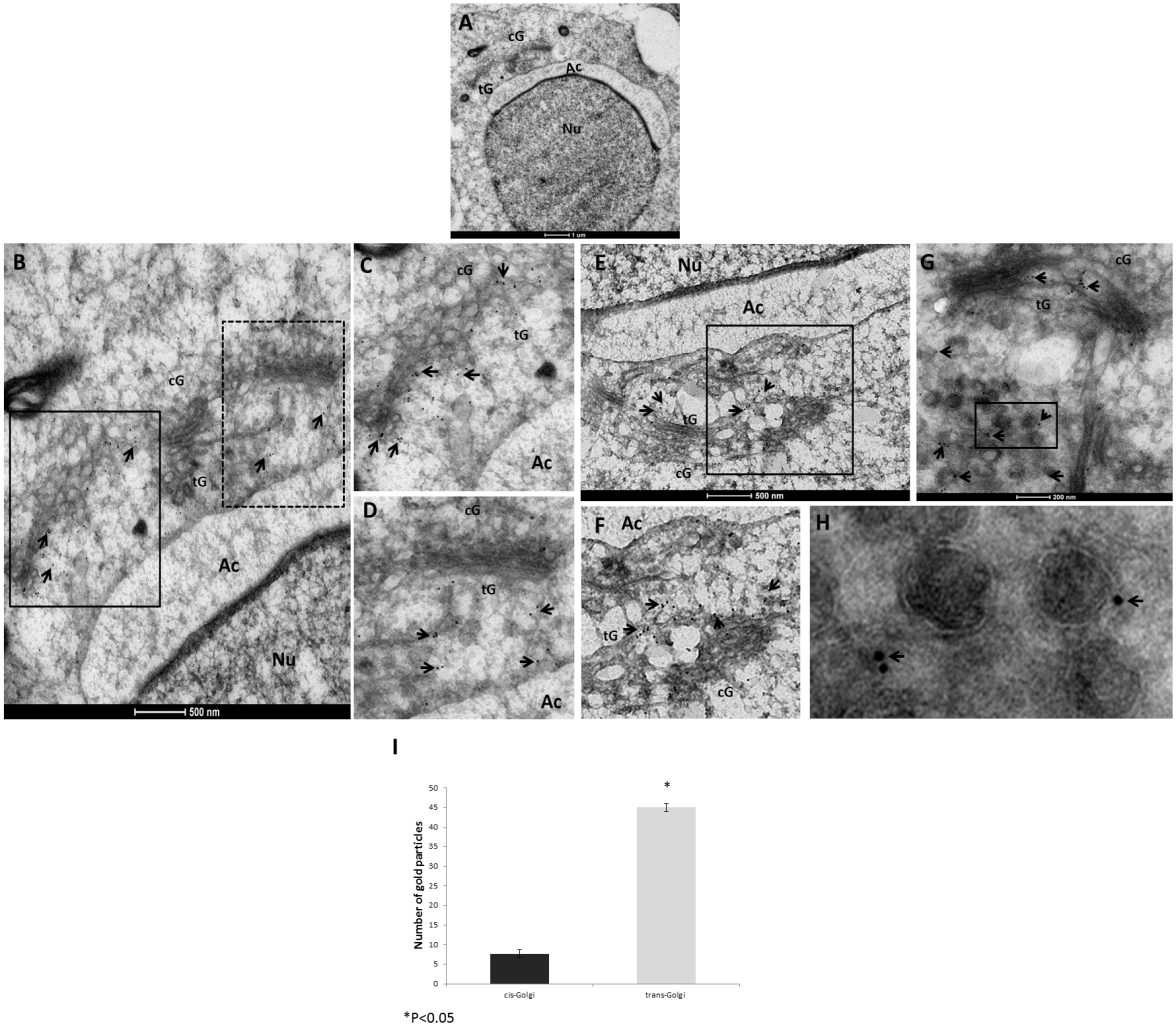
TMF resides in the trans-Golgi compartment of stages 6–8 round spermatids. Testicular sections prepared as described were subjected to IG-EM analysis using anti-TMF specific antibody followed by an anti-rabbit secondary antibody conjugated to gold particles with a diameter of 10nm (black dots). (A) Stage 6 spermatids. (B) The same spermatid from **A** under higher magnification showing the positive staining of TMF, which is marked by arrows, in the trans-Golgi and the space between the Golgi and the acrosome. (C) Enlarged boxed area in **B**. Positive staining of TMF is marked by arrows. (D) Enlarged dashed-boxed area in **B**. Positive staining of TMF is marked by arrows. (E) A stage 7 spermatid showing the same trans-Golgi localization of TMF, which is marked by arrows. (F) Enlarged boxed area in **E**. TMF staining is marked by arrows. (G) Stage 8 spermatid showing trans-Golgi and association of TMF along with vesicles association (marked by arrows). (H) Enlarged boxed area in G. Arrows mark TMF associated with the vesicles outer membrane. cG = cis-Golgi, tG = trans-Golgi, Ac = acrosome, Nu = nucleus. Bars represent 1μm (A), 500nm (B-F) and 200nm (G-H). Each image represents one out of twelve different cells selected from three different sections which gave similar results. (I) Comparative quantification of the immuno-gold labeled TMF in the different Golgi compartments. n = 5 (different cells of the same stage from three different sections), histograms represent mean values +/- SD.

We previously described the localization of TMF to the Golgi of spermatocytes and round spermatids, and its importance in acrosome formation [3, 4]. To more precisely characterize the localization of TMF inside the Golgi during spermiogenesis, we performed Immunogold staining of TMF followed by electron microscopic analysis (IG-EM) of mice testicular sections. Using testicular sections prepared from 10 weeks old wild type (wt) mice, this approach enabled us to determine the sub-organelle localization of TMF in Golgi compartments of developing round spermatids. The Golgi apparatus structure is well defined in round spermatids with a horseshoe shape and a cis-Golgi in the base of the shape facing the outer cell membrane and a trans Golgi network facing the nucleus [31]. The IG-EM experiments revealed the specific accumulation of TMF in the cis-Golgi compartment of round spermatids at stages 3 and 4, undergoing the "Golgi-phase" of acrosome development. During these early stages, TMF accumulated preferentially in the periphery of the cis-Golgi cisterns (Fig 1A–1F, marked by arrows in B-E). Notably, when the same sections were examined for the localization of TMF in later stage round spermatids, during the "cap-phases" of acrosome development, TMF was mostly found in the trans-Golgi network and in the inter-space, lying between the acrosome and the Golgi apparatus. In contrast to stages 3–4, TMF was hardly detected in the cis-Golgi compartment of stages 6 and 7 spermatids (Fig 2A–2F, marked by arrows in B-F). When staged 8 round spermatids were examined in the stained sections, TMF was localized mainly to the surface of vesicles derived from the trans-Golgi network, and to the trans-Golgi (Fig 2G and 2H, marked by arrows). Notably, TMF localization was confined to the external surface of the vesicle membranes, and not to the inner side of the membranes (Fig 2H, marked by arrows). Quantification of the TMF positive staining in the different Golgi compartments revealed that TMF associates mainly with the trans-Golgi compartment in round spermatids staged at stages 6–8 (Fig 2I). Notably, in spermatocytes which do not undergo acrosome biogenesis, TMF was co-localized with both cis and the trans-Golgi markers, suggesting that in these cells it is spread throughout the Golgi stacks without a specific sub-localization (S1 Fig). These results depict the dynamic localization of TMF in the different Golgi compartments, trans-locating from the cis-Golgi after the first stages of spermiogenesis to the trans-Golgi and vesicular membrane at later stages of spermatid development.

### TMF is required for proper spatial orientation of the Golgi and for CB formation during sperm development

**Fig 3:**
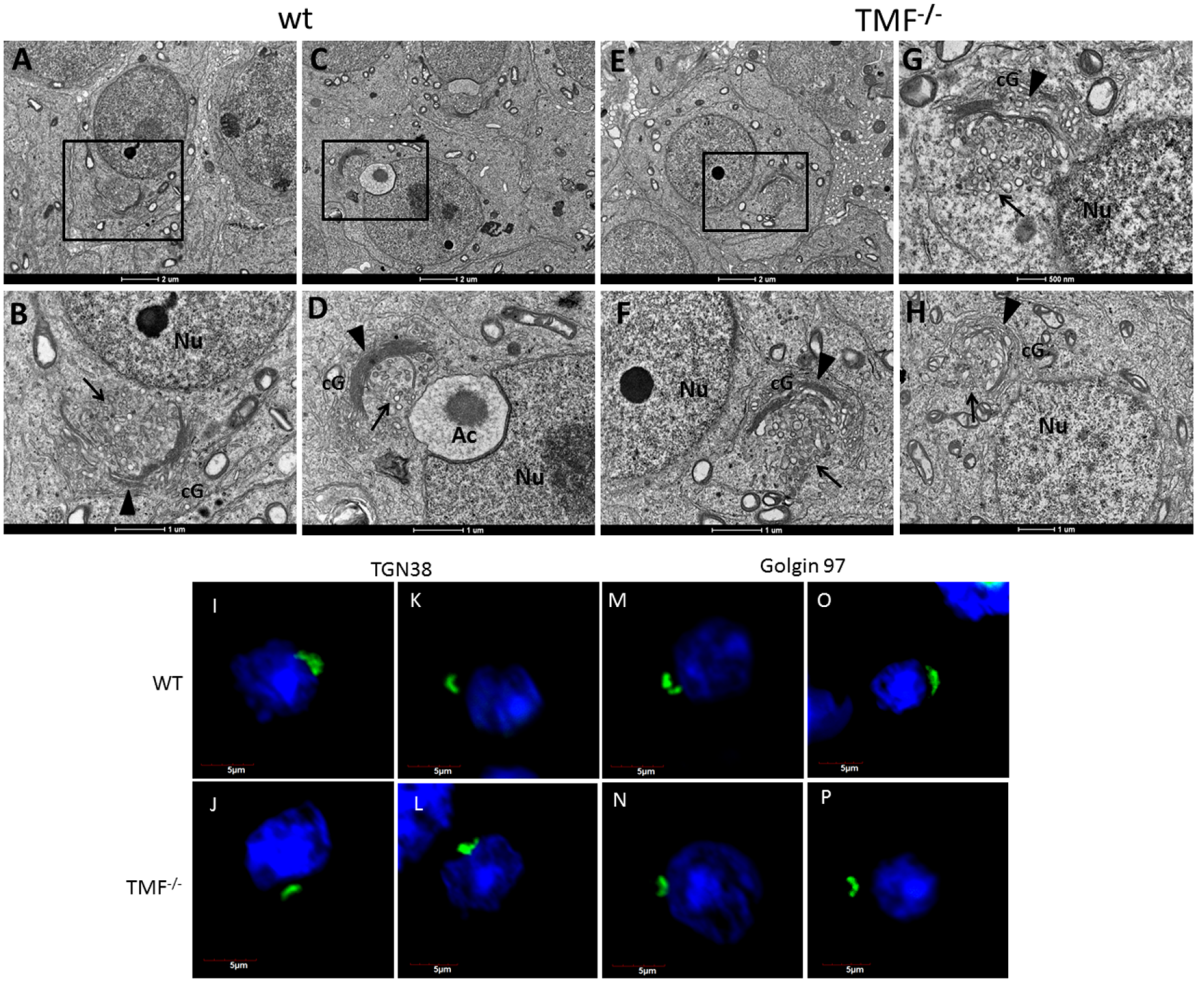
Abnormal Golgi orientation in TMF^-/-^ spermatids. TEM analysis of testicular sections from wt (A-D) and TMF^-/-^ mice (E-H). (A) Stage 1 wt spermatid. (B) Boxed area in **A** under higher magnification. The Golgi apparatus is marked by an arrow head. Pro-acrosomal vesicles are marked by an arrow. (C) Stage 3 wt spermatid. (D) Boxed area in **C** under higher magnification. The Golgi apparatus is marked by an arrow head. Pro-acrosomal vesicles are marked by arrow. (E) Stage 3 TMF^-/-^ spermatid. (F) Boxed area in **E** under higher magnification. The Golgi apparatus is marked by an arrow head. Pro-acrosomal vesicles are marked by an arrow. (G) Different TMF^-/-^ round spermatids of the same stage as in **E**. The Golgi apparatus is marked by an arrow head. Pro-acrosomal vesicles are marked by an arrow. (H) Stage 1 TMF^-/-^ spermatid. The Golgi apparatus is marked by an arrow head. Pro-acrosomal vesicles are marked by an arrow. Ac = acrosome, Nu = Nucleus, cG = cis-Golgi. Bars represent 2μm (A,C and E), 1μm (B, D, F and H) and 500 nm (G). Each image represents one out of twenty different cells selected from five different sections which gave similar results. Immunofluorescence staining of the trans-Golgi marker TGN38 in wt (I, K), and TMF^-/-^ round spermatids (J, L) (Green). Immunofluorescence staining of the trans-Golgi marker Golgin 97 in wt (M and O), and TMF^-/-^ round spermatids (N, P) (Green). Nuclei were visualized with Hoechst (Blue). Bars represent 5 μm.

**Fig 4:**
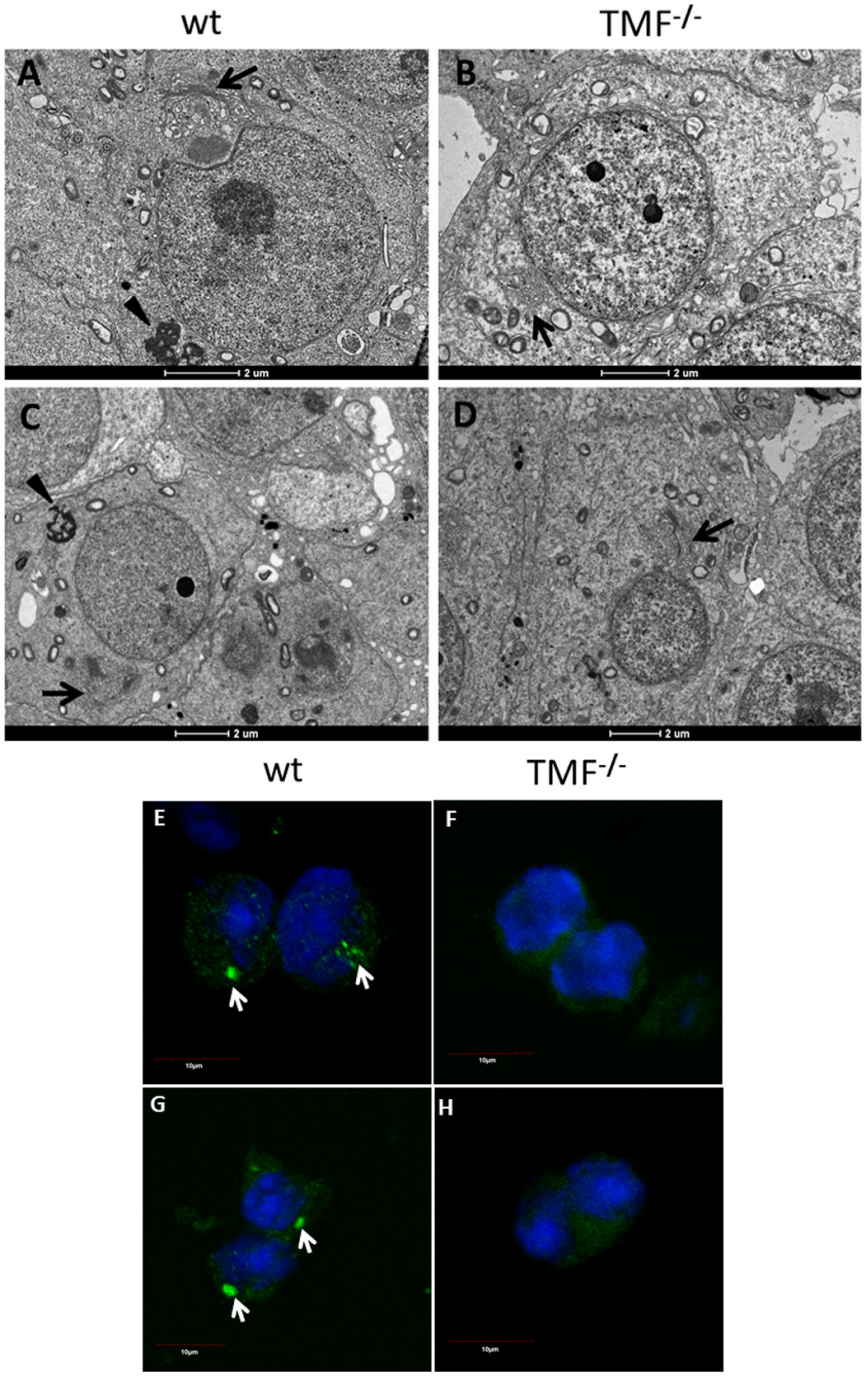
TMF^-/-^ round spermatids lack the CB compartment. Testicular sections from wt (A and C) or TMF^-/-^ (B and D) mice were subjected to TEM analysis. (A) Stage 4 round spermatid of wt mice. The Golgi apparatus is marked by an arrow. The CB is marked by an arrow head. (B) Stage 4 TMF^-/-^ round spermatid. The Golgi is marked by an arrow. No CB is observed in this spermatid. (C) Early stage 1 spermatid of wt mice. The Golgi is marked by an arrow. The CB is marked by an arrow head. (D) TMF^-/-^ spermatid of an early stage 1. The Golgi is marked by an arrow. No CB is observed in this spermatid. Bars represent 2μm. (E and G) Testicular round spermatids of wt mice were subjected to KIF17B immunofluorescence staining using specific antibodies (green, aggregations of KIF17B are marked by arrows). (F and H) TMF^-/-^ testicular round spermatids were subjected to KIF17B immunofluorescence staining using specific antibodies (green). Nuclei were visualized with Hoechst. Bars represent 10μm.

**Fig 5:**
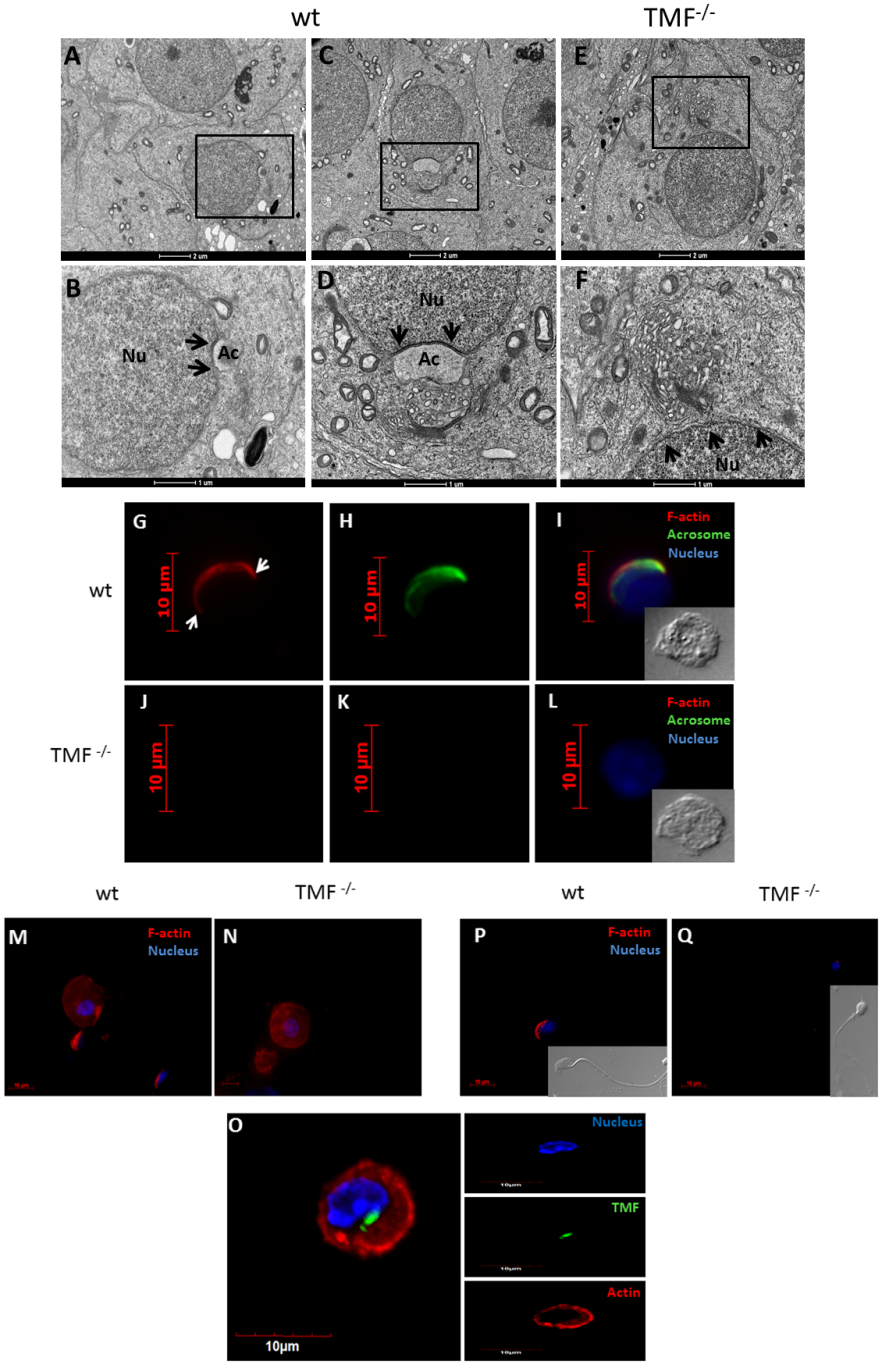
TMF^-/-^ spermatids lack an acroplaxome. Testicular sections from wt (A-D) and TMF^-/-^ mice (E-F) were subjected to EM analyses. (A) Stage 2/early 3 wt spermatid. (B) Boxed area in **A** under higher magnification. The acroplaxome is marked by arrows. (C) Stage 4 wt spermatid. (D) The boxed area in **C** under higher magnification. The acroplaxome is marked by arrows. (E) Stage 4 TMF^-/-^ spermatid. (F) The boxed area in **E** under higher magnification. The supposed localization of the acroplaxome is marked by arrows. Ac = acrosome, Nu = Nucleus. Bars represent 2μm (A, C and E) and 1μm (B, D and F). Each image represents one out of twenty different cells selected from three different sections which gave similar results. Immunocytochemical staining of F-actin (red) and acrosome (green) in round spermatids from wt (G-I) and TMF^-/-^ (J-L) mice, which were exposed to hypotonic shock.DIC images of the stained spermatids are shown in **I** and **L**. Acroplaxome margins are marked by arrows in **G**. (M) Immunocytochemical staining of F-actin (red) in wt round spermatid. (N) Immunocytochemical staining of F-actin (red) in TMF^-/-^ round spermatid. (O) Immunocytochemical staining of F-actin (red) and TMF (Green) in wt round spermatid. (P) Immunocytochemical staining of F-actin (red) in wt elongated spermatid. (Q) Immunocytochemical staining of F-actin (red) in TMF^-/-^ elongated spermatid. DIC images of the spermatids are also shown in **P** and **Q**. Nuclei were visualized with Hoechst solution (blue). Bars represent 10μm. Images represent one out of five independently prepared cell suspensions that gave similar staining profiles.

The dynamic and stage dependent altered localization of TMF in the Golgi compartments during the progression of spermiogenesis, suggested the involvement of this protein in controlling the dynamic and altered spatial orientation of the Golgi during sperm development. We therefore examined the integrity and orientation of the Golgi during spermiogenesis, using transmission electron microscopy (TEM) of TMF mutant mice testes. This analysis revealed that unlike wt round spermatids in which the trans-Golgi compartment faces the cell nucleus (Fig 3A–3D (marked by arrow head in B and D), and Figs 4A and 5C and 5D), TMF^-/-^ round spermatids bear an abnormal spatial orientation of the Golgi apparatus, in which the trans-Golgi compartment faces the cytoplasm (Fig 3E–3H, cis-Golgi marked by an arrow heads in F-H). Vesicles derived from the trans-Golgi were seen in both the wt (Fig 3B and 3D marked by arrows) and TMF^-/-^ round spermatids (Fig 3F–3H, marked by arrows). However, while wt vesicles were observed in the vicinity of their presumed target, the acrosome (Fig 3B and 3D), in the TMF^-/-^ cells, the Golgi-derived vesicles seemed to lack homing and trafficking guidance towards the cell nucleus (Fig 3F–3H). To further corroborate the abnormal Golgi orientation in TMF^-/-^ round spermatids, an immuno-fluorescence staining of trans-Golgi markers was carried out in these cells. While wt spermatids exhibited a typical horseshoe shaped Golgi facing the nucleus (Fig 3I, 3K, 3M and 3O), in TMF^-/-^ spermatids the Golgi appeared in its normal horseshoe shape but its trans-compartment deviated and was facing an opposite orientation to the nucleus (Fig 3J, 3L, 3N and 3P). This complies with the images seen in the TEM images of TMF^-/-^ spermatids. Thus, TMF is required for maintaining proper spatial orientation of the Golgi during sperm development. Another anomaly which was noticed in TMF^-/-^ spermatids was the lack of chromatoid body (CB). While stage 1 and 4 wt round spermatids contained native CB structure as seen by TEM analysis (Fig 4A and 4C, CB marked by arrow heads), in TMF^-/-^ spermatids The CB structure was not observed (Fig 4B and 4D). The lack of CB in TMF^-/-^ round spermatids was also manifested by the immuno-staining of KIF17B, a protein which normally aggregates in the CB and is responsible for RNA trafficking from the nucleus to the CB [32]. This immuno-staining analysis revealed both, the dispersion of KIF17B throughout the cell and its aggregation around the nucleus in wt round spermatids, as was previously described [32] (Fig 4E and 4G, aggregate marked by an arrow). However, in TMF^-/-^ round spermatids KIF17B was found only in its dispersed pattern and not in its aggregated form around the nucleus. Thus, corroborating the absence of CB in TMF^-/-^ spermatids (Fig 4F and 4H).

### TMF^-/-^ spermatids lack the acroplaxome scaffold infrastructure

The absence of both acrosome structure and proacrosomal vesicles from the nuclear envelope of TMF^-/-^ spermatids could also reflect lack of the acroplaxome scaffold in these cells. We therefore used TEM to examine the acroplaxome structure in wt and TMF^-/-^ spermatids. While wt round spermatids exhibited a normal acroplaxome overlaying the nuclear envelope, with typical density and structure (Fig 5A–5D, marked by arrows in B and D), TMF^-/-^ round spermatids were devoid of any acroplaxome structure and showed abnormal Golgi orientation (Fig 5E and 5F, marked by arrows in F).

To further corroborate the lack of acroplaxome structure in TMF^-/-^ spermatids, we analyzed the presence of a typical, perinuclear F-actin sheet, which is part of the acroplaxome [7]. WT and TMF^-/-^ spermatids from which the cytoplasm was depleted by hypotonic shock, were stained with phalloidin for F-actin detection. This staining revealed the perinuclear presence of F-actin in wt round spermatids, overlapping acrosome staining in these cells (Fig 5G–5I marked by white arrows in G). In comparison, no staining of either peri-nuclear F-actin or the acrosome was seen in TMF^-/-^ spermatids (Fig 5J–5L), supporting the notion that TMF^-/-^ round spermatids also lack the acroplaxome structure. It should be noted that while the perinuclear F-actin that assembles the acroplaxome was compromised in TMF^-/-^ spermatids, their cytoskeletal F-actin structure remained normal, as compared to wt spermatids (Fig 5M and 5N). Co-staining of TMF and F-actin in wt round spermatids revealed the normal actin organization around the Golgi and throughout the cytoplasm without proximal localization of TMF to actin fibers (Fig 5O). When later stages of spermatids (stage 12–15) were subjected to F-actin immunocytochemical analysis, F-actin surrounding the sperm cells heads was normally seen in wt mice (Fig 5P), but was not detected in the heads of the TMF^-/-^ spermatids from the same stages, in which only faint staining for these filaments could be observed accompanied by spherical shaped head of these cells (Fig 5Q).

### TMF associates with both tubulin and microtubules

**Fig 6:**
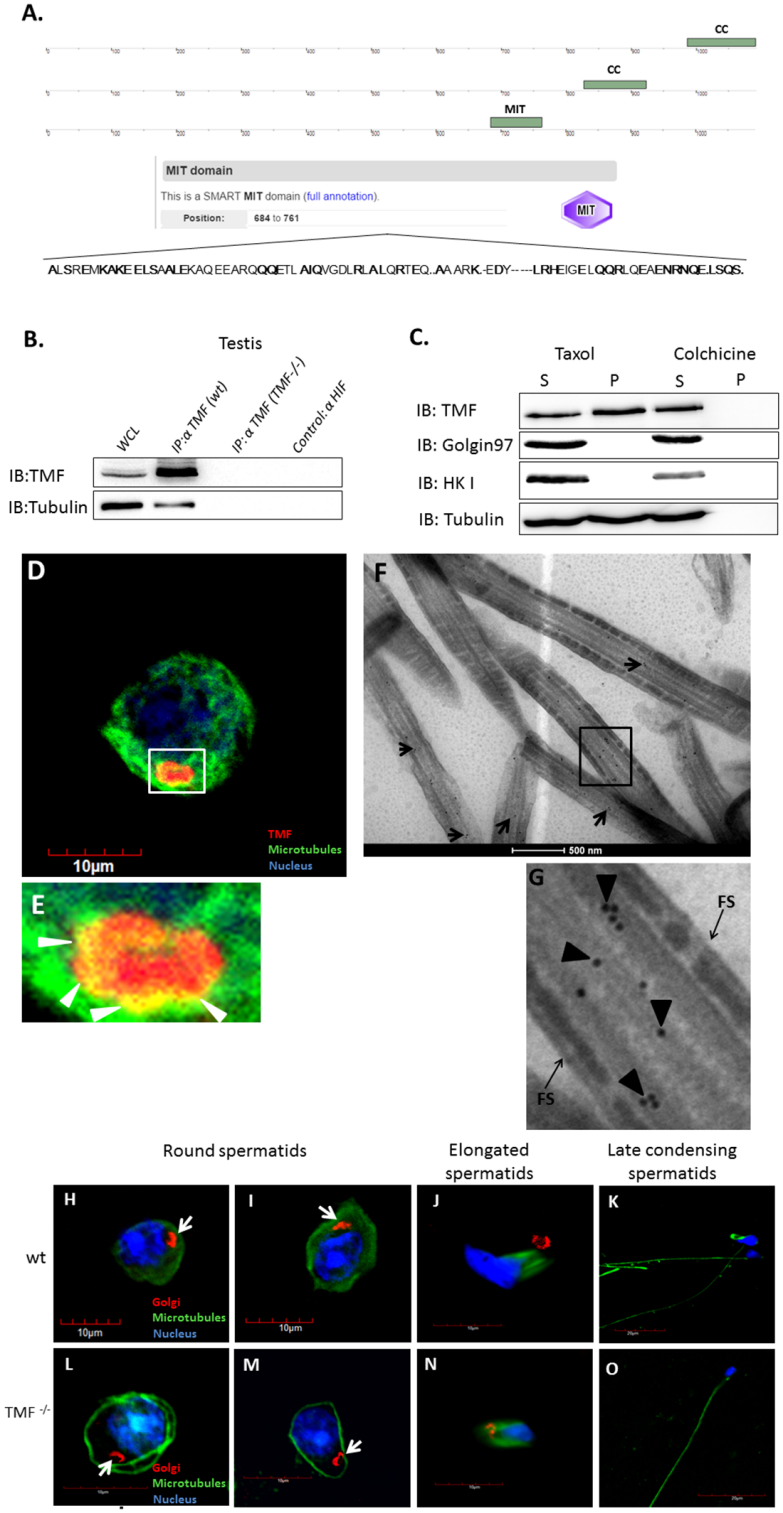
TMF contains an MIT domain and associates with tubulin and microtubules. (A) The protein sequence of TMF was analyzed using the SMART algorithm. CC = coiled-coil. MIT = microtubule interacting domain. Underneath the motifs location scheme is the MIT sequence of TMF and its exact location inside TMF protein sequence. In bold are the key preserved amino acids of the sequence which are identical to the consensus MIT sequence. (B) Protein extracts from wt and TMF^-/-^ mice testes were subjected to immuno-precipitation (IP) analysis using anti-TMF antibodies followed by western-blot analysis to detect tubulin co-precipitation. WCL = whole cell lysate from wt testes, which is the IP input. (C) Co-precipitation of microtubules and TMF from mice spermatogenic cells. S denotes the supernatant which contains all the soluble proteins. P represents the precipitate which contains the sedimented microtubules. (D) Immuno-fluorescence co-staining of TMF (Red) and microtubules (Green) in round spermatid. (E) Enlarged boxed area in **D**. Co-localization of TMF and microtubules (Yellow) is depicted by white arrow heads. Nuclei were visualized with Hoechst solution (blue). Bar in **D** represents 10μm. (F) IG-EM analysis of TMF (black dots, presented by arrows) carried-out on testicular sections from wt mouse. (G) Enlarged boxed area in **F**. TMF positive staining is indicated by arrow heads. The FS is indicated by arrows. Bar represents 500nm. Immunocytochemical analysis of the Golgi marker GM130 (red) and microtubules (green) in wt (H-K) and TMF^-/-^ spermatids (L-O). The Golgi apparatus is marked by arrows in H-I and L-M. Nuclei were visualized with Hoechst solution (blue). Bars represent 10μm (H-J and L-N) and 20μm (K and O). Images represent one out of four independently prepared cell suspensions that gave similar staining profiles.

The involvement of TMF in the maintenance of proper Golgi orientation in spermatogenic cells raised the possibility that TMF not only associates with the Golgi, but also with cytoskeletal filaments which were shown to direct the anchoring of this organelle [16, 33]. As an initial step, we applied an *in-silico* search for known motifs in the mouse TMF aa sequence using the "Simple Modular Architecture Research Tool" (SMART, http://smart.embl-heidelberg.de/) algorithm. This algorithm enables the identification of known functional domains that are contained in a given protein sequence. Our analysis revealed the presence of two coiled-coil (CC) domains in the C-terminal part of TMF (Fig 6A). These domains were described previously and were shown to be required for attaching TMF to the Golgi apparatus [34]. In addition to these motifs, a novel, microtubule interacting domain (MIT) was identified near the C-terminal region of TMF (Fig 6A). To experimentally verify the ability of TMF to associate with microtubules and tubulin, several approaches were adopted. Lysates from mouse testes were immuno-precipitated using affinity purified TMF antibodies, and the co-immuno-precipitation of tubulin and TMF was examined using western-blot analysis. TMF^-/-^ testicular extract was used as a negative control for the co-immuno-precipitation along with beads which were conjugated with different primary antibody directed towards HIF. This analysis revealed the co-association of TMF and tubulin in wt testicular lysates (Fig 6B). To substantiate the ability of TMF to associate with microtubules, we performed a precipitation of intact microtubules from mice spermatogenic cells along with their associated proteins. These analyses clearly demonstrated that TMF, but not HK I or golgin97 which served as negative controls, co-precipitated along with microtubules (Fig 6C). However, when spermatogenic cells were subjected to Colchicine which disrupts assembled microtubules TMF was not precipitated, thus substantiating the notion that TMF associates and co-precipitates with microtubules (Fig 6C). To further corroborate the association of TMF with microtubules in spermatogenic cells we co-immuno-stained these cells for microtubules and TMF. These experiments demonstrated the specific co-localization of TMF and microtubules around the presumed periphery of the Golgi apparatus (Fig 6D and 6E, co-localization marked by arrow heads in E). Furthermore, IG-EM analysis carried out on testicular sections from wt mice demonstrated the localization of TMF to a well characterized microtubules structure and to the outer dense fiber (ODF) region in the spermatid flagella (Fig 6F and 6G, marked by arrows in F and arrow heads in G) but not to the peripheral fibrous sheath (FS) (Fig 6F, marked by arrows). After confirming the ability of TMF to associate with tubulin and microtubules, we examined whether absence of TMF can affect microtubule organization around the Golgi apparatus in spermatogenic cells. WT and TMF^-/-^ spermatogenic cells were subjected to immunocytochemical analysis using anti-tubulin and anti-GM130 (a Golgi marker) antibodies. This staining revealed a deformation in the microtubules structure around the Golgi in TMF^-/-^ round spermatids when compared to the wt structure. While in wt round spermatids, the microtubules were spread throughout the cytoplasm and reached the outer side of the Golgi apparatus (Fig 6H and 6I, marked by an arrow), in the TMF^-/-^ round spermatids, the microtubules demonstrated axial positioning without spreading in the cytoplasm and without achieving close proximity to the Golgi, which by itself was characterized by abnormal spatial orientation (Fig 6L and 6M, marked by arrows), as seen also in the EM analysis (Fig 3F–3H). The tubulin dynamics occurring at later stages of spermiogenesis, which are also characterized by the exclusion of the Golgi apparatus (stages 9–16), appeared normal in TMF^-/-^ elongated spermatids as compared to the wt cells (Fig 6J, 6K, 6N and 6O).

### The MIT domain of TMF is crucial for its localization to the Golgi apparatus

**Fig 7:**
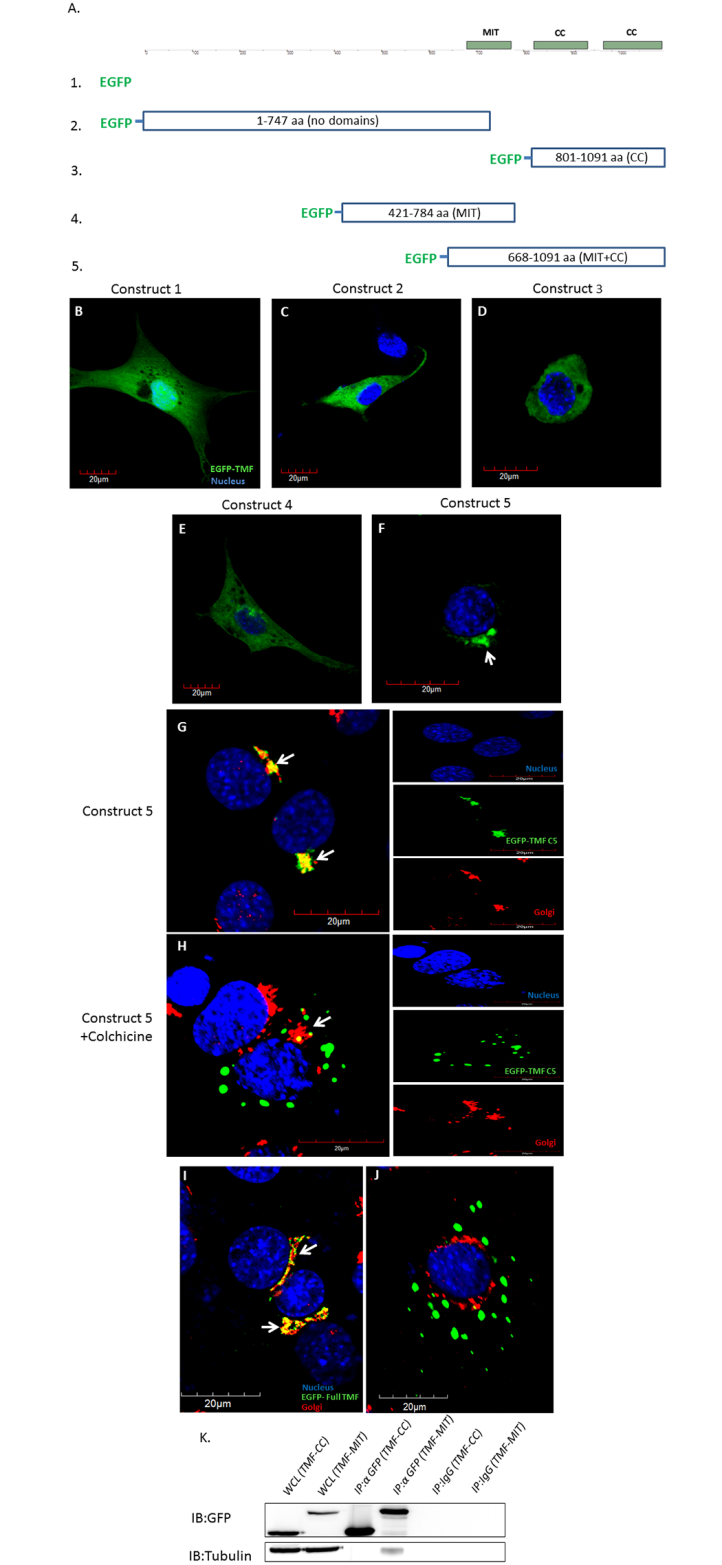
CC and MIT domains are required for stable association of TMF with the Golgi. (A) Diagram of the TMF aa sequence with the marked MIT and CC motifs (top). Constructs 1–5 transfected into NIH3T3 cells are depicted below. The TMF aa included in each construct are shown, and the presence of the CC or MIT motifs in each construct are marked in the brackets. (B-F) Confocal microscopy analysis of EGFP-fused TMF segments, ectopically expressed (green) in NIH3T3 cells. (G) Confocal microscopy analysis of TMF segment containing the MIT and c-terminus CC domain fused to EGFP (EGFP-TMF C5) (green) overlapping the immuno-staining of GM130 (Golgi marker, red.). Arrows represent co-localization (yellow color). The different fluorescence channels are presented in the right panels. Bar represents 20μm. Nuclei were visualized with Hoechst solution (blue). (H) As in **G**, only that these cells were treated for 20 min with colchicine. Arrow indicates the Golgi apparatus. Images represent typical fluorescence and staining profiles obtained for all transfected cells in four independent transfection experiments. (I) Confocal microscope analysis of the full TMF protein fused to EGFP (green) overlapping the immuno-stained Golgi (golgin 97- red.) (J) As in **I**, only that cells were treated for 20 min with colchicine. Arrow indicates the Golgi apparatus. Nuclei were visualized with Hoechst solution (blue).Images represent typical fluorescence and staining profiles obtained for all transfected cells in four independent transfection experiments. (K) IP of GFP-TMF protein fragments from lysates of NIH3T3 cells expressing either GFP-TMF containing the MIT domain (TMF-MIT) or GFP-TMF fragment containing the CC domains without the MIT motif (TMF-CC).Whole cell lysate (WCL) represents the input of the IP. IgG beads served as a negative control. The IP was followed by a WB analysis to assess the co-IP of the TMF fragment and tubulin. Images represent one out of five independent typical WB analyses which gave similar results.

After corroborating the ability of TMF to associate with tubulin and microtubules, and since microtubules have a great impact on Golgi apparatus localization and functionality [35], we reasoned that TMF may simultaneously associate with microtubules and the Golgi apparatus. This would imply that in addition to the known C-terminal CC domains, the MIT domain may also be important for stable association of TMF with the Golgi apparatus. To substantiate this idea, NIH3T3 mouse fibroblasts were transfected with different constructs bearing segments of TMF fused to the green fluorescent protein (EGFP) (Fig 7A). Inspection of the transfected cells by confocal microscopy revealed the cytoplasmic dispersion of all TMF constructs that lacked the mutual presence of the C-terminal CC domains and the MIT motif (Fig 7B–7E). The only construct that exhibited a confined, peri-nuclear localization was the TMF segment, containing both the MIT domain and the C-terminal CC domains (construct 5) (Fig 7F). To verify that the peri-nuclear localization reflects a Golgi association of the exogenous TMF, the transfected cells were immuno-stained with antibody specifically recognizing the Golgi apparatus. This analysis confirmed that the localization of this TMF segment is confined to the Golgi (Fig 7G, marked by arrows). Immunostaining for microtubules in these cells transfected with construct 5 also revealed the co-localization of the exogenous TMF with microtubules (S2A Fig). To directly examine the stabilizing effect of microtubules on the association of TMF with the Golgi, cells transfected with construct 5 were treated for 20 min with the microtubules disrupting agent-Colchicine [36]. This led to the disruption of the microtubules architecture and disengagement of the TMF segment from the Golgi apparatus without scattering the organelle itself (Fig 7H, S2B and S2C Fig). To further corroborate that the dispersion of the TMF fragment after brief (20 min) Colchicine treatment correlates with dis-integration of the microtubules rather than with fragmentation of the Golgi organelle we subjected NIH3T3 cells to co-immuno-staining of microtubules and the Golgi. This analysis revealed that untreated cells display a normal microtubules structure which also co-localizes with the Golgi apparatus (S2D Fig). However, exposure of cells to Colchicine for 20 min led to the disintegration of microtubules while the Golgi apparatus still exhibited an intact peri-nuclear structure (S2E Fig). After 40 min of Colchicine treatment the microtubules architecture was completely compromised and the Golgi apparatus was scattered and no longer possessed a typical packed peri-nuclear localization (S2F Fig).Thus, the scattering of the ectopic construct 5- TMF, after a brief Colchicine treatment, is related to the disassembly of microtubules. To exclude the possibility that the microtubules dependent localization of the truncated TMF- construct 5 to the Golgi, does not reflect a specific behavior exerted only by the fragmented TMF protein, we also transfected NIH3T3 cells with a vector encoding a EGFP-fused full length TMF. The cells were then immuno-stained for the Golgi apparatus. This revealed the co-localization of the intact TMF protein with the Golgi (Fig 7I). Similarly to cells expressing the TMF-construct 5, 20 min of Colchicine treatment dispersed the ectopically expressed intact TMF (Fig 7J). To substantiate the role of the MIT domain, in mediating the association of TMF with tubulin, the ability of this motif to co-precipitate with tubulin was examined in NIH3T3 cells which were transfected with vectors expressing either fragment 3 or 5 of the TMF protein (Fig 7A). Co-immuno-precipitation analysis revealed that only fragment 5 which harbors the MIT domain was able to associate and to co-precipitate with tubulin (Fig 7K).

## Discussion

TMF has been previously shown by us to reside in the Golgi of spermatogenic cells and to be essential for acrosome formation and normal development of spermatids and spermatozoa [3]. In this study, we further deciphered the regulatory processes through which TMF ensures the proper development and maturation of sperm cells. We found that the location of TMF in the Golgi of developing spermatids is dynamic, and is altered throughout the spermiogenic developmental process. In spermatocytes, TMF co-localized with cis or trans- Golgi markers and appeared to be dispersed throughput the Golgi stacks. When spermatids at stages 3 and 4, residing at the Golgi-phases [37] where inspected for TMF localization, TMF appeared to localize to the cis-Golgi compartment. Moreover, TMF is primarily found in the peripheral sites of the cis-Golgi compartment, which were shown to interact with microtubules [38]. The early steps of spermiogenesis are characterized by re-orientation of the Golgi towards the cell nucleus, and tethering of trans-Golgi-derived vesicles onto the acroplaxome to initiate acrosome formation in round spermatids [31, 39]. The cis-Golgi region does not bear the capacity to release Golgi-derived vesicles to the cytoplasm but is rather engaged in tethering vesicles emerging from the ER, or releasing vesicles back to the ER [40, 41]. In addition, the cis-Golgi also serves as an interacting platform with cytoskeletal microtubules, an interaction that is mediated through defined protein adaptors [42]. Localization of TMF to the periphery of the cis-Golgi rear surface during early spermiogenic steps, and the abnormal orientation of the Golgi in TMF^-/-^ round spermatids, suggests the involvement of this protein in the spatial re-orientation of the Golgi at these early stages. We propose that TMF helps to achieve the proper spatial orientation of the Golgi, which must be established early in spermiogenesis, thereby enabling emergence of Golgi-derived vesicles toward the spermatid nuclear envelope. Cytoskeletal fibers such as microtubule filaments are capable of connecting to the Golgi apparatus, and support its ability to undergo intra-cellular dynamics and movement [15]. Furthermore, when the microtubules architecture becomes compromised in somatic and in spermatogenic cells, the Golgi apparatus loses its structure and functionality [43, 44]. Hence, we propose a model in which TMF with its MIT domain and microtubules association capacity may support the establishment of a defined spatial orientation of the Golgi in round spermatids, by serving as a mediator between microtubules and the organelle. This suggested role of TMF is substantiated by the presence of both CC and MIT domains in the protein. CC sequences are important for golgins docking and attachment to the Golgi apparatus [45], and the MIT domain enables the association of proteins with microtubules [46]. Indeed, the link between the MIT domain and the association of TMF with tubulin was demonstrated through both the co-IP of tubulin and the GFP-TMF fragment containing the MIT element, from transfected NIH3T3 cells and the co-IP of TMF and tubulin from testicular lysates. Furthermore, the association of TMF with microtubules in spermatogenic cells was confirmed using three independent approaches. We used a sensitive IG-EM analysis to reveal the localization of TMF to the well characterized microtubules structure and the outer dense fibers (ODF) which immobilize several tubulin interacting proteins, at the sperm flagella [47, 48]. An immuno-fluorescence co-staining of spermatogenic cells for both TMF and microtubules revealed the co-localization of TMF and microtubules in the periphery of the presumed cis-Golgi. This profile resembles the localization of another cis-Golgi residing golgins like 58K, which bear the capacity of associating with microtubules [45, 49]. Finally, a co-precipitation analysis of microtubules and their associated proteins from spermatogenic cells, clearly demonstrated the association of TMF with these cytoskeletal filaments. Thus, early in spermiogenesis, TMF may associate with the cis-Golgi and with microtubules, thereby allowing the Golgi to reach its proper orientation. This proposed model is supported also by our findings that the presence of both, the previously described CC sequences [50] and the newly characterized MIT motif in TMF, are both required, along with microtubules integrity, for stable association of TMF with the Golgi. This most probably reflects the necessary interaction of TMF with both the microtubules and the Golgi apparatus.. Unlike our results the TMF C-terminal portion, which bears only CC motifs without the MIT, was previously shown to localize to the Golgi apparatus [51]. However, in this published work an highly over-expression system of TMF has been applied (an SV40 based vector expressed in COS cells) [51], turning it incomparable to our current analysis which was carried out using a non-replicating vector in NIH3T3 cells. Additional evidence for the involvement of TMF in Golgi docking to microtubules stems from the fact that TMF^-/-^ round spermatids bear an abnormal distribution of microtubules which unlike seen in normal spermatogenic cells, lack proximal positioning to the Golgi apparatus [38, 44]. Notably, these TMF^-/-^ cells also clearly exhibit an abnormal Golgi orientation but yet a normal architecture. Thus, unlike observed in somatic cells [51], in spermatogenic cells TMF seems to govern the Golgi orientation rather than the Golgi integrity. Our suggested function of TMF, in mediating Golgi-tubulin interactions, appears transient and is restricted to the early spermiogenic stages round spermatids, when the reversed orientation of the Golgi toward the nucleus is initially established, and the formation of a new organelle, the acrosome, is initiated. However, after these early spermiogenic stages the secretion and proper targeting of vesicles toward the acrosome is crucial for the complete development of this organelle [39, 52]. At these later spermiogenic stages TMF trans-locates from the cis-Golgi compartment to the trans-Golgi network and vesicle membrane, thereby supporting our suggested function of TMF which is required for establishing proper Golgi orientation only during early stages of spermiogenesis. The trans-Golgi is important for vesicle secretion and it regulates their targeted trafficking during spermiogenesis [52, 53]. The presence of TMF in the trans-Golgi network and on the vesicles membrane during late spermiogenesis, as shown for the first time in this study, suggests an additional role for this protein during spermatogenesis, which is more related to vesicle trafficking and targeting.. The fact that TMF is present in pro-acrosomal vesicles but not on the acrosome itself, suggests that TMF completes its task once the pro-acrosomal vesicle reaches the acrosome. While the lack of TMF compromises Golgi orientation during the first stages of spermiogenesis, pro-acrosomal vesicles still emerge from the TMF^-/-^ trans-Golgi network. However, these vesicles fail to properly home towards the nucleus of round spermatids, thereby failing to form the acrosome structure [52], corroborating with the notion that in later staged spermatids TMF is needed for the proper targeting of vesicles. This observation portrays TMF as a vesicles guiding protein and it could coincide with the reported involvement of TMF in vesicles retrograde trafficking from the endosome to the Golgi and from the Golgi to the endoplasmic reticulum in somatic cells [54]. During the later stages of spermiogenesis, after the complete development of the acrosome, the Golgi apparatus is excluded from the developing sperm cells, and the Golgi-tubulin linking function of TMF seems to no longer be required. This notion is based on the observation reported in our current study that, although the microtubule alignment with the Golgi apparatus is perturbed in TMF^-/-^ round spermatids, its dynamic shaping appears to be normal in elongated spermatids and spermatozoa where the Golgi apparatus is no longer needed and it begins its guided movement to the caudal side of the spermatid in order to be excluded [11, 24]. This is reflected by the proper transient structure of the manchette, and later in the normal tubular flagella in TMF^-/-^ sperm [3].

The specific abnormal organization of microtubules in TMF^-/-^ round spermatids can also compromise the formation of the CB organelle. The CB serves as a main RNA processing center which is found at various stages of the developing spermatids. Formation of the CB and its regulated motility were shown to depend on microtubules integrity. This is manifested by the fact that microtubules disrupting agents cause disintegration of the CB therefore directly linking the integrity of this organelle to intactness of microtubules [55]. Notably, it was also shown that vesicles emerged from the Golgi are found in the CB vicinity and are incorporated into this organelle. This, suggest that Golgi derived vesicles contribute to the CB formation and function. [32, 56, 57]. Thus, abnormal microtubules structure and the impaired vesicles trafficking in TMF^-/-^ spermatids seem to compromise the CB formation in these cells.

Besides targeting pro-acrosomal vesicles toward the nucleus, spermatids need to overlay their nuclear envelope with the acroplaxome, which serves as a tethering scaffold for the pro-acrosomal vesicles [23]. Although the detailed formation process of the acroplaxome has not been revealed, this structure was shown to consist mainly of F-actin and keratin 5 [7]. Lack of normal acroplaxome was seen in sperm of mice devoid of the acrosomal associated protein SPACA-1, and this led to the development of abnormal spermatids which lack the formation of functional acrosome [58]. Notably, TMF^-/-^ spermatids not only lack an acrosome, but are also completely devoid of the acroplaxome. TMF^-/-^ spermatids are the first reported spermatids to lack vesicle homing, acrosome and acroplaxome structure, and to bear an abnormal Golgi spatial orientation. Other published gene knockouts for Golgi or pro-acrosomal vesicles associated proteins, bearing abnormal spermatids such as the GOPC^-/-^, Hrb^-/-^ Galnt^-/-^, or PICK1^-/-^ showed a failure of acrosome formation, but exhibited normal vesicle trafficking, normal Golgi structure and orientation, and presence of acroplaxome structure with normal F-actin [25, 26, 59, 60]. Thus, the lack of acroplaxome in TMF^-/-^ spermatids could be linked to the impaired homing of Golgi-derived vesicles in these cells, and may suggest that acroplaxome formation is also driven by Golgi-derived vesicles.

Our results show here for the first time that a single Golgi associated protein is required for both Golgi orientation and vesicle targeting during an intricate developmental process like spermiogenesis.

## Supporting Information

S1 Fig TMF co-localizes with both the cis and trans-Golgi compartments in spermatocytes. Spermatocytes were extracted from mice testes and were immuno-stained for TMF (Green), the cis-Golgi marker 58K (Red) (A), or the trans-Golgi marker Golgin97 (Red) (B). Separate channels are presented to the right of each merged image. The boxed area in each merged image is enlarged and presented. Bars represent 20μm.
(TIF)

S2 Fig Localization of the construct 5-TMF to the Golgi apparatus depends on microtubules integrity. (A) Confocal microscope analysis of NIH3T3 cells transfected with the TMF fragment containing the MIT and c-terminus CC domain fused to EGFP (EGFP-TMF C5) (green) and immuno-stained for microtubules (red). Boxed area is enlarged and is presented in the top right corner of the image. Arrows indicate co-localization (yellow color). (B and C) The same as in **A** only that these cells were treated with Colchicine for 20 and 40 min respectively. Images represent typical fluorescence and staining profiles obtained from all transfected cells in four independent transfection experiments. (D) NIH3T3 cells were co -immuno-stained for the Golgi (red) and microtubules (green). Boxed area is enlarged and presented in the top right corner of the image. Arrows indicate co-localization (yellow color). (E-F) The same as in **D** only that cells were treated with Colchicine for 20 and 40 min respectively. Nuclei were visualized using staining with Hoechst (blue).
(TIF)

S1 Table Primers used for constructing the EGFP-TMF chimeric constructs. The numbers depict the amino acids (a.a) comprising each TMF segment. In brackets,(+) denotes the inclusion of the indicated domain, and (–) denotes the absence of the domain. CC = coiled-coil domain. MIT = microtubule interacting domain.
(TIF)
