# Supplementary figures and images for: TMF/ARA160 Governs the Dynamic Spatial Orientation of the Golgi Apparatus during Sperm Development
Source: PLoS One. 2015 Dec 23;10(12):e0145277. doi: 10.1371/journal.pone.0145277 (PMC4689540; doi:10.1371/journal.pone.0145277)

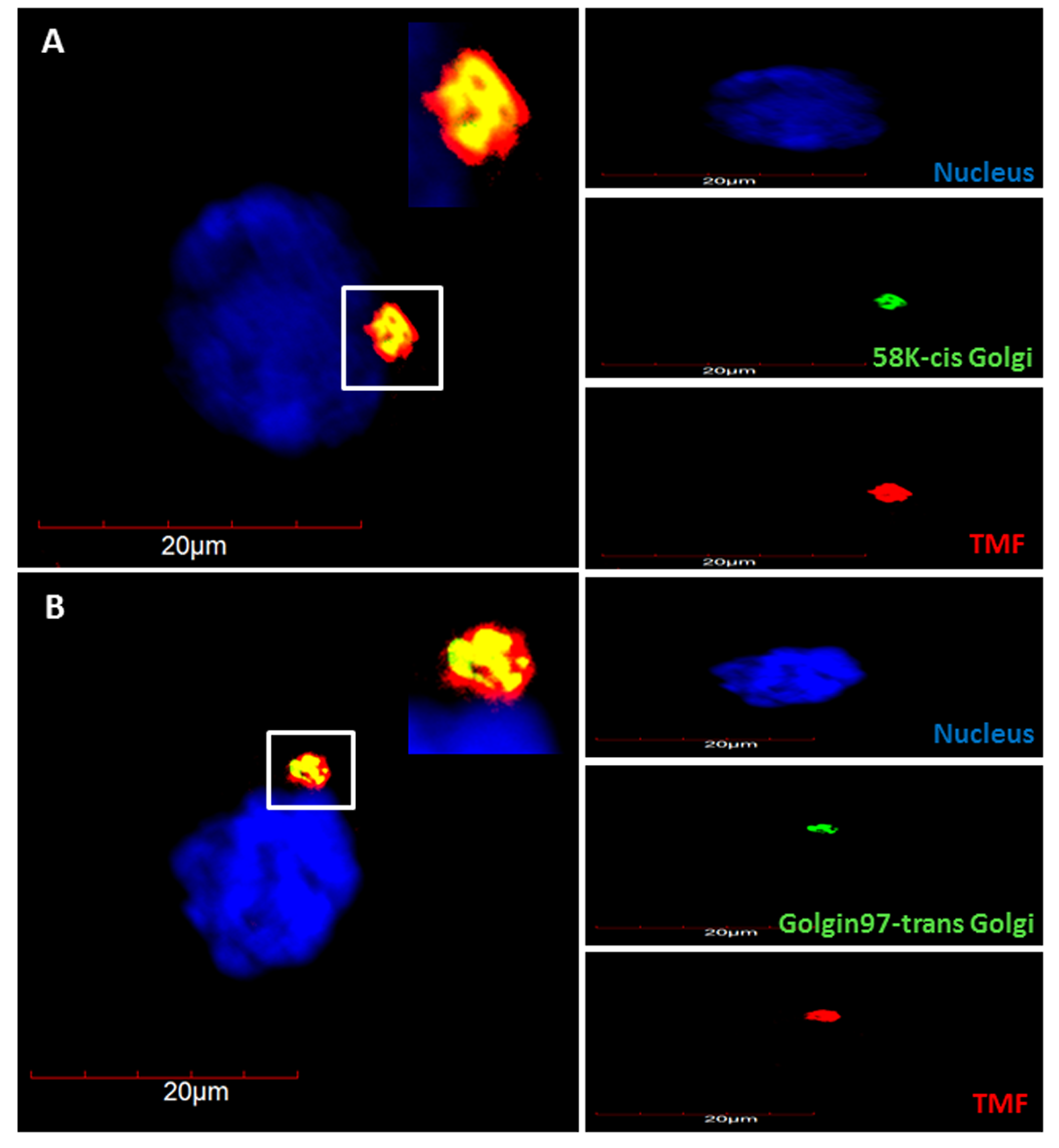

Supplement: S1 Fig — Spermatocytes were extracted from mice testes and were immuno-stained for TMF (Green), the cis-Golgi marker 58K (Red) (A), or the trans-Golgi marker Golgin97 (Red) (B). Separate channels are presented to the right of each merged image. The boxed area in each merged image is enlarged and presented. Bars represent 20μm. (TIF) [file pone.0145277.s001.tif]

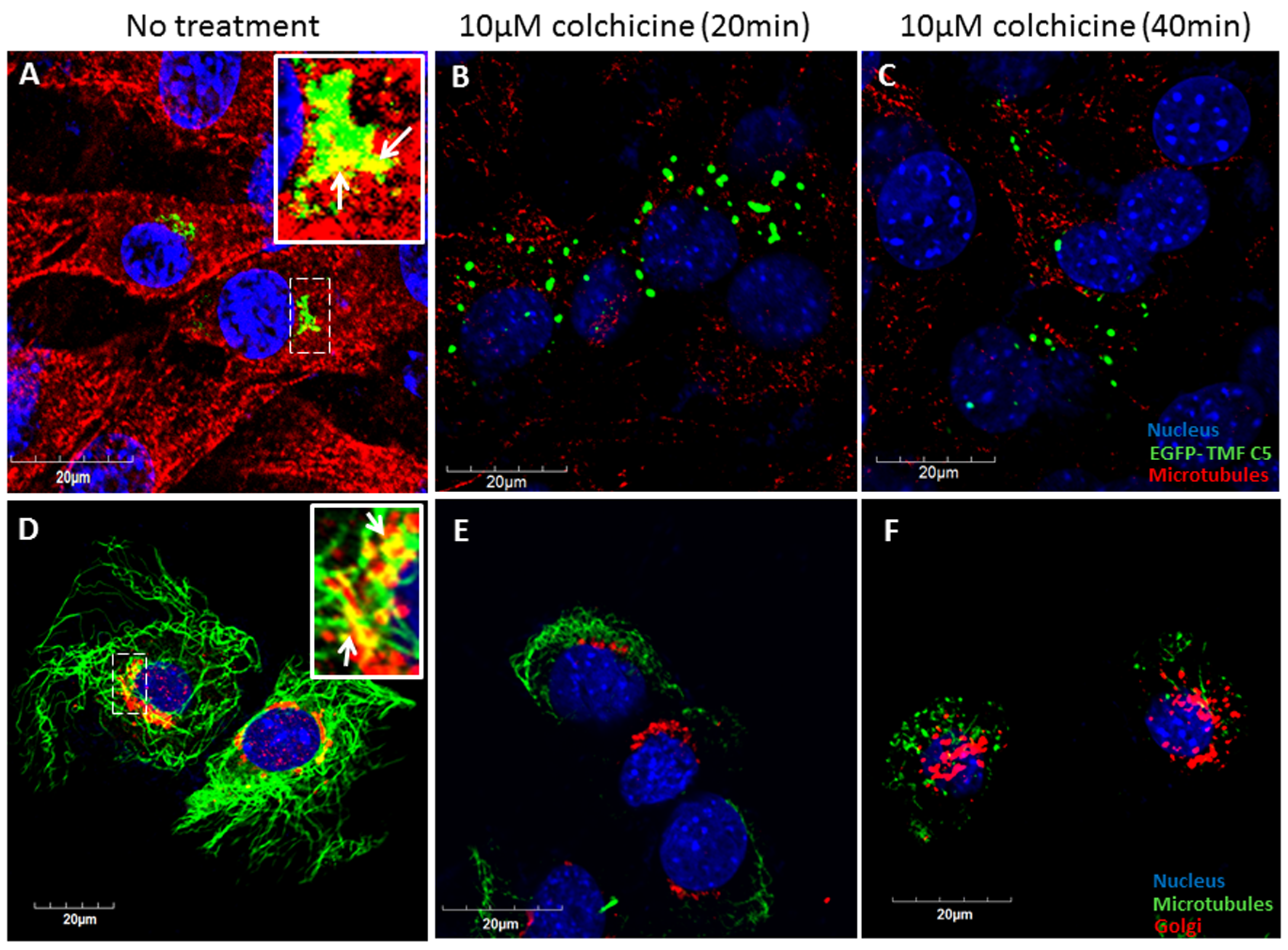

Supplement: S2 Fig — (A) Confocal microscope analysis of NIH3T3 cells transfected with the TMF fragment containing the MIT and c-terminus CC domain fused to EGFP (EGFP-TMF C5) (green) and immuno-stained for microtubules (red). Boxed area is enlarged and is presented in the top right corner of the image. Arrows indicate co-localization (yellow color). (B and C) The same as in A only that these cells were treated with Colchicine for 20 and 40 min respectively. Images represent typical fluorescence and staining profiles obtained from all transfected cells in four independent transfection experiments. (D) NIH3T3 cells were co -immuno-stained for the Golgi (red) and microtubules (green). Boxed area is enlarged and presented in the top right corner of the image. Arrows indicate co-localization (yellow color). (E-F) The same as in D only that cells were treated with Colchicine for 20 and 40 min respectively. Nuclei were visualized using staining with Hoechst (blue). (TIF) [file pone.0145277.s002.tif]

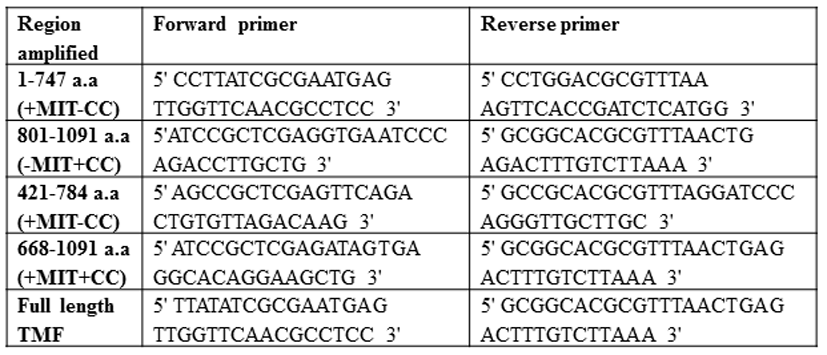

Supplement: S1 Table — The numbers depict the amino acids (a.a) comprising each TMF segment. In brackets,(+) denotes the inclusion of the indicated domain, and (–) denotes the absence of the domain. CC = coiled-coil domain. MIT = microtubule interacting domain. (TIF) [file pone.0145277.s003.tif]
